# Randomised, Double-Blind, Placebo-Controlled Study of Iguratimod in the Treatment of Active Spondyloarthritis

**DOI:** 10.3389/fmed.2021.678864

**Published:** 2021-06-02

**Authors:** Yan Li, Kunpeng Li, Zheng Zhao, Yanyan Wang, Jingyu Jin, Junhua Guo, Jie Zhang, Jianglin Zhang, Jian Zhu, Feng Huang

**Affiliations:** ^1^Medical School of Chinese People's Liberation Army, Beijing, China; ^2^Department of Rheumatology and Immunology, First Medical Center, General Hospital of Chinese People's Liberation Army, Beijing, China

**Keywords:** Iguratimod, spondyloarthritis, efficacy, safety, treatment

## Abstract

**Background and Purpose:** The effect of Iguratimod in the treatment of rheumatoid arthritis was confirmed in past studies. In terms of the mechanism of the effect and clinical application experience, Iguratimod has a potential value in the treatment of spondyloarthritis (SpA). This study evaluated the efficacy and safety of Iguratimod on active SpA.

**Methods:** Subjects with active SpA were enrolled and randomly divided into two groups at a ratio of 1:2 (placebo vs. Iguratimod). On the basis of non-steroidal anti-inflammatory drugs, combined treatment with Iguratimod or placebo, followed by follow-up every 4 weeks for 24 weeks. The primary efficacy endpoint was to evaluate the alleviation rate of ASAS20; the important improvement of ASDAS and the efficacy of spinal mobility, physical function and quality of life at the 24th week.

**Results:** A total of 48 cases in the Iguratimod group and 25 cases in the placebo group were included in the final analysis. On the 24th week, the percentage of responders to ASAS20 (80 vs. 44%) and ASAS40 (56 vs. 20%) treated with Iguratimod were significantly higher than that in the placebo group (*P* < 0.05). Twelve cases had gastrointestinal discomfort, of which eight were in the Iguratimod group (16.7%, one case withdrew from the study due to diarrhoea) and four were in the placebo group (16.0%). No significant difference was found between the two groups (*P* < 0.05). Three cases of elevated transaminase were observed in the Iguratimod group and none in the placebo group, with no significant difference (*P* < 0.05).

**Conclusion:** Iguratimod could significantly reduce the symptoms and signs of patients with active SpA. It could improve the physical function and quality of life of these patients and the overall safety and tolerance are good.

## Highlights

- The efficacy of Iguratimod in the treatment of rheumatoid arthritis was confirmed. This study proved that the drug could be used for the treatment of active spondyloarthritis and as a new clinical treatment option.

## Introduction

Spondyloarthritis (SpA) is a group of systemic inflammatory diseases that mainly affect the axial spine, peripheral joints, and entheses, and it could eventually lead to ligament ossification and bony rigidity. It is mainly manifested as enthesitis and synovitis, often invading the spine and large joints of the lower extremities and causing disability; it could also be accompanied by various comorbidities, such as sarcopenia, bone loss, and metabolic abnormalities (1). Ankylosing spondylitis (AS) is the prototype of SpA. Its prevalence in China is ~0.3–0.5%, and it usually develops in adolescence. It is a disease with high incidence and disability. The treatment of SpA can be divided into non-drug therapy and drug therapy (2–4). Non-drug therapy mainly includes disease education, exercise and massage, ultrasound, and hyperthermia (5). Disease education and exercise are the two cornerstones of non-drug therapy. At present, EULAR and ACR recommendations for the management of SpA only recommend non-steroidal anti-inflammatory drugs (NSAIDs), tumour necrosis factor-α inhibitors and fully human IL-17A monoclonal antibodies as approved drugs for the treatment of AS. They have not yet approved other slow-acting drugs to be used for treating AS. Most doctors also use sulfasalazine, methotrexate, and thalidomide to treat AS on the basis of their own clinical experience and with varying curative effects.

Iguratimod, also known as T-614, is a new type of small molecule compound with anti-inflammatory and immunomodulatory effects; It was listed in China (2011) and Japan (2012) for the treatment of rheumatoid arthritis; its safety and effectiveness have been verified in patients with rheumatoid arthritis (6). As Iguratimod could inhibit the production of inflammatory cytokines, such as IL-1 and TNF; block the IL-17 signalling pathway and inhibit cyclooxygenase (7), Iguratimod may be effective in the treatment of SPA/AS. However, no rigorous clinical research exists to confirm this speculation. Therefore, this study aimed to evaluate the efficacy and safety of Iguratimod in patients with active SpA.

## Methods

### Research Objects and Design

Patients with active SpA who were diagnosed and treated in our centre from January 2017 to June 2019 were included in this single-centre, randomised, double-blind, placebo-controlled trial (Registration No: ChiCTR-IPR-15006753). Before randomisation was conducted, a 4-week screening period was used to assess eligibility, and then a 24-week treatment was conducted. The eligible patients with SpA filled in the medical record report form and were followed up six times in 24 weeks for pain symptoms, spinal joint function, life function, imaging examination and laboratory examination after Iguratimod administration. The study protocol was approved by the Ethics Committee of the PLA General Hospital (Approval No: S2014-111-01) and all patients signed an informed consent form. For sample-size estimation, this study used the ASAS20 response rate as the main research indicator. In accordance with the literature, the ASAS20 response rates in the control and Iguratimod groups were ~30 and 65%, respectively. In addition, α was set to 0.05, whilst β was set to 0.20. The sample ratio of the control and Iguratimod groups was 1:2, and their calculated sample sizes were n1 = 21 and n2 = 42, respectively, that is, the control group had 21 cases and the Iguratimod group had 42. Considering a 15% drop, the sample size of the control group was set to 25 cases, whilst that of the Iguratimod group was set to 50.

### Inclusion and Exclusion Criteria

The inclusion criteria were as follows: subjects aged 18–65 years who met the New York diagnostic criteria for AS revised in 1984 or the 2009 ASAS axial SpA classification criteria and those in the screening and baseline periods. The activity status is defined as meeting at least two of the following three conditions: (1) bath AS disease activity index (BASDAI) score ≥ 4; (2) in the visual analogue scale (VAS), total back pain VAS ≥ 4 cm; and (3) morning stiffness ≥ 1 h (2–4). The exclusion criteria were as follows: patients currently receiving corticosteroid therapy and traditional DMARDs or within 3 months, patients who used various traditional Chinese medicine preparations in the past, with a withdrawal time of < 3 months, patients who used TNFi and anti-IL17; patients with other rheumatic autoimmune diseases other than SpA; patients whose disease was accompanied by any of the following: ALT, AST exceeding 1.5 times the upper limit of normal, serum creatinine greater than the upper limit of normal and WBC < 3 × 10^9^/L or HGB < 85 g/L or PLT < 100 × 10^9^/L; patients with severe cardiovascular, kidney and other important organ, blood and endocrine system diseases, malignant tumours and medical history; pregnant women, lactating women and men, or women planning to conceive in the near future and patients with immunodeficiency, uncontrolled infections, and active gastrointestinal tract disease.

### Methods

A total of 75 patients were enrolled and randomly divided into Iguratimod group (50 cases) and controlled group (25 cases), with a ratio of 2:1. By using the block randomisation method, the random number was generated by statistical professionals through the SAS software analysis system to generate a continuous serial number equivalent to the sample size, that is, the drug number. The researcher provided the corresponding drugs for treatment in the order of the subjects' enrolment. One group received NSAIDs + two 25 mg Iguratimod/day of treatment, and the other group received NSAIDs + placebo treatment. In the course of this study, all patients took a stable and sufficient amount of NSAIDs (aximethacin sustained-release capsules or meloxicam tablets). On this basis, a placebo was selected as a control, not only meeting the ethical requirements but also effectively prevented trial bias and systematic errors. Iguratimod was given to the Iguratimod group twice a day at 25 mg each time, whilst the placebo control group received tablets imitating Iguratimod (with no pharmacological effects) twice a day at one tablet each time.

### Study Endpoints

Efficacy and safety assessments were conducted during the baseline period and every 4 weeks thereafter until the end of the study. The primary efficacy endpoint was to achieve the percentage of patients achieving the ASAS20 response and a clinically important improvement at 24th week. The ASAS20, ASAS40, partial remission and ASAS5/6 in the ASAS efficacy evaluation are defined as follows: ① patient's global assessment of disease activity (PtGA 0–10 cm VAS), ② night back pain and total back pain (0–10 cm VAS), ③ Bath AS Functional Index (BASFI), ④ inflammatory / morning stiffness (mean question 5 and 6 of the BASDAI), ⑤ CRP (mg/L) and ⑥ spinal mobility [lateral lumbar flexion from Bath AS Metrology Index (BASMI)]. In ASAS20, compared with the baseline data, the improvement of ≥one point in 3 of the 4 domains + No worsening of ≥20% + ≥1 (0–10) in domains. In ASAS40, compared with the baseline data, the improvement of ≥2 points in 3 of 4 domains + No worsening in any domain. The one item that failed to achieve 40% improvement did not deteriorate compared with the baseline. For partial alleviation, ①–④ items score ≤ two points. In ASAS 5/6, compared with the baseline data, the improvement of at least five domains in ①–⑥ is ≥20%. The AS Disease Activity Score (ASDAS) is a recently developed composite measure incorporating questions 2 (neck, back or hip pain), 3 (joint pain/swelling), and 6 (duration of morning stiffness) of the BASDAI, the PtGA, and hs-CRP. Using the ASDAS, patients were categorised into the following disease state categories: inactive (ASDAS score < 1.3), moderate (≥1.3– < 2.1), high (≥2.1– ≤ 3.5), and very high (>3.5). Clinically important improvement was defined as a decrease from baseline in the ASDAS of ≥1.1, and major improvement was defined as a decrease from baseline in the ASDAS of ≥2.0. Safety assessments were conducted at each study visit and included adverse event monitoring and clinical laboratory and vital sign evaluation. SF-36 and ASASHI scorings were performed at the end of 24 weeks of treatment.

### Statistical Methods

Consecutive variables were expressed as mean ± SD and category variables were expressed as “*n* (%)”. For the comparison of baseline consecutive variables between the two groups, independent-sample *t* test was used when the data obeyed a normal distribution and the homogeneity of variance was satisfied. Otherwise, the Wilcoxon rank sum test was used. The category variables between the two groups was compared using *X*^2^ test or Fisher's exact test. The comparison of consecutive variables between the two groups before treatment and at 12 and 24 weeks after treatment was analysed using mixed effect models for repeated-measure design analysis of variance, with Bonferroni method for multiple comparisons. The comparison of category variables between the two groups before treatment and at 12 and 24 weeks after treatment was analysed using generalised estimating equations. The Bonferroni method was used to correct *P* values for multiple comparisons. All statistical analyses were conducted using SAS software and the difference was considered statistically significant when *P* < 0.05.

## Results

### Baseline Characteristics

**Figure 1:**
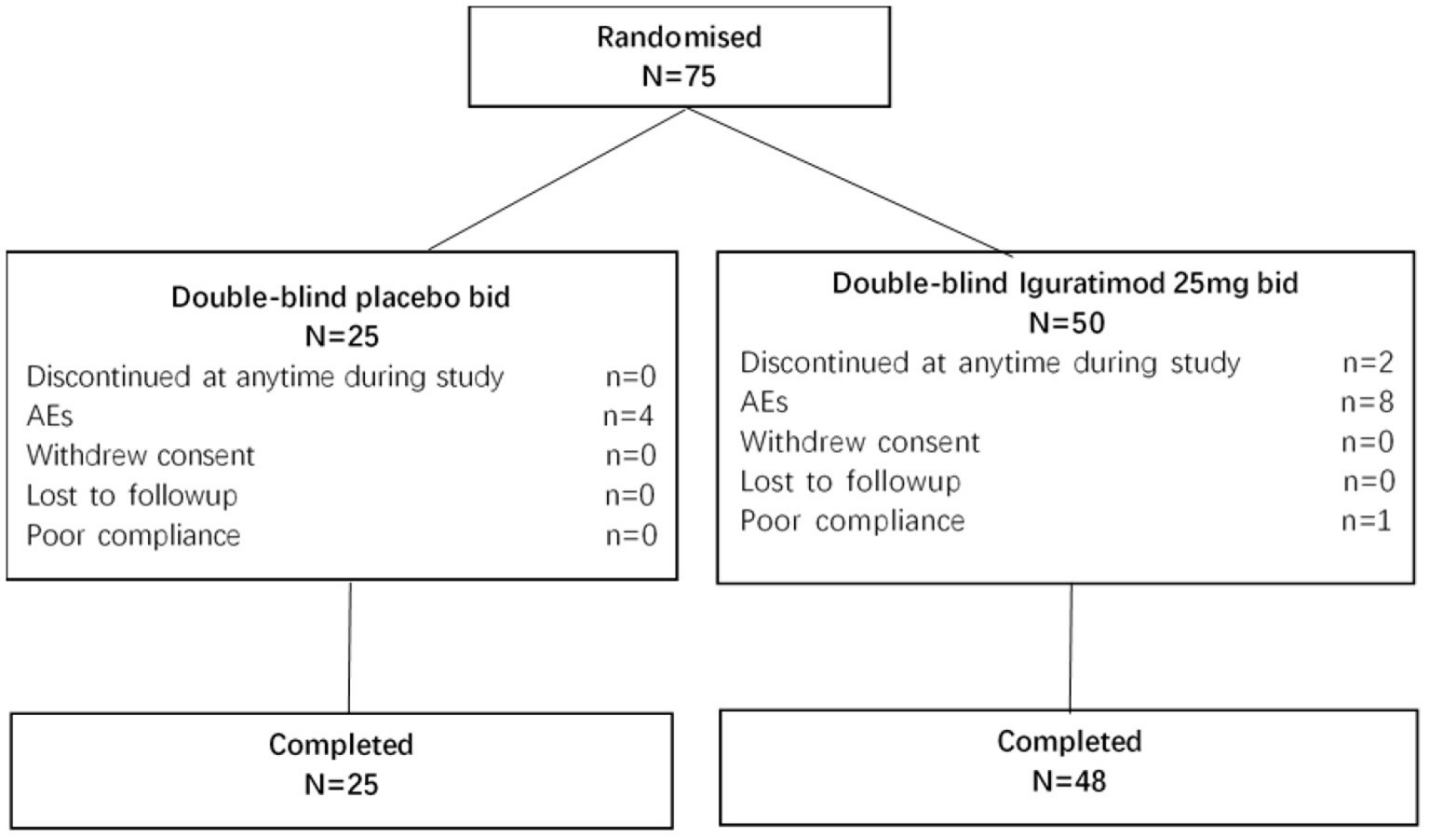
Patient disposition.

This study included 75 study subjects, of which two (in the Iguratimod group) were withdrawn from the study and 73 were completed (25 in the control group and 48 in the Iguratimod group), which was in line with the sample requirements of 21 cases in the control group and 42 cases in the Iguratimod group (Figure 1). Tables 1, 2 show the comparison of demographic and medical history data between the two groups. No statistical difference between the two groups was found (*P* > 0.05).

**Table 1:** Comparison of baseline data between the two groups.

**Characteristics**	**Controlled group (*n* = 25)**	**Iguratimod (*n* = 48)**	**t/X^**2**^**	***P value***
Male (*n*, %)	23 (92.00)	39 (81.25)	1.4844	0.2231
Age (years)	30.28 ± 5.94	31.38 ± 7.36	0.4254	0.6706
Course of disease (years)	6.44 ± 4.57	7.73 ± 6.87	0.3267	0.7439
Age of onset (years)	23.36 ± 5.51	23.27 ± 8.45	0.0408	0.9675
Night back pain	6.28 ± 0.98	6.52 ± 1.03	−0.9895	0.3224
Waist pain (*n*, %)	13 (52.00)	26 (54.17)	0.0310	0.8602
Neck pain (*n*, %)	4 (16.00)	3 (6.25)	-	0.2218
Chest and back pain (*n*, %)	4 (16.00)	6 (12.50)	-	0.7274
Hip joint pain (*n*, %)	9 (36.00)	16 (33.33)	0.0519	0.8198
Uveitis (*n*, %)	1 (4.00)	2 (4.17)	-	1.0000
Heel pain (*n*, %)	2 (8.00)	8 (16.67)	-	0.4776
Peripheral joint pain (*n*, %)	2 (8.00)	5 (10.42)	-	1.0000
Anterior chest wall pain (*n*, %)	1 (4.00)	8 (16.67)	-	0.1521
Symphysis pubis pain (*n*, %)	0 (0.00)	2 (4.17)	-	0.5434
Ischial tubercle pain (*n*, %)	1 (4.00)	4 (8.33)	-	0.6545
Family history (*n*, %)	7 (28.00)	15 (31.25)	0.0825	0.7740
Smoking (*n*, %)	15 (60.00)	26 (54.17)	1.1953	0.5501
Alcohol (*n*, %)	12 (48.00)	28 (58.33)	0.7086	0.3999
BMI	24.43 ± 3.41	24.60 ± 4.14	−0.1736	0.8627

**Table 2:** Comparison of effectiveness indicators between the two groups of patients baseline and after 24 weeks treatments (quantitative indicators).

**Parameters**	**Baseline**			**24weeks**		
	**CG (*n* = 25)**	**IG (*n* = 48)**	***P***	**CG (*n* = 25)**	**IG (*n* = 48)**	***P***
ASDAS	3.05 ± 0.51	3.24 ± 0.57	1.0000	2.30 ± 0.76	1.49 ± 0.71	< 0.0001
BASDAI	4.57 ± 0.57	4.69 ± 0.94	1.0000	3.04 ± 0.96	1.33 ± 0.81	< 0.0001
BASFI	3.49 ± 1.23	3.41 ± 1.33	1.0000	2.06 ± 1.15	1.15 ± 0.78	0.0100
BASMI	2.44 ± 1.87	2.21 ± 2.02	1.0000	1.92 ± 1.80	1.56 ± 1.76	1.0000
Night back pain	6.28 ± 0.98	6.52 ± 1.03	1.0000	3.80 ± 1.12	1.58 ± 1.29	< 0.0001
General back pain	5.44 ± 0.92	5.90 ± 0.81	0.4212	4.04 ± 1.06	2.04 ± 1.18	< 0.0001
PtGA	5.92 ± 0.91	6.06 ± 0.86	1.0000	3.80 ± 0.96	1.79 ± 1.20	< 0.0001
PGA	5.56 ± 0.77	5.90 ± 0.66	0.8190	3.96 ± 1.06	1.90 ± 1.08	< 0.0001
Platelet (× 10^9^/L)	327.56 ± 85.62	300.58 ± 70.55	0.7930	314.56 ± 64.98	278.19 ± 68.58	0.2622
ESR (mm/h)	29.88 ± 18.56	27.06 ± 16.39	1.0000	18.92 ± 17.38	15.21 ± 14.69	1.0000
CRP (ug/L)	13.52 ± 14.02	14.59 ± 14.11	1.0000	12.21 ± 14.97	6.81 ± 10.85	0.6212
Duration of morning stiffness (hours)	4.52 ± 2.63	4.56 ± 2.53	1.0000	2.04 ± 1.46	0.83 ± 1.02	0.1002
Lateral flexion	12.24 ± 6.02	11.89 ± 6.22	1.0000	13.6 ± 6.26	13.29 ± 5.91	1.0000
Tragus to wall distance	15.12 ± 3.78	14.63 ± 3.88	1.0000	14.76 ± 3.97	13.81 ± 3.11	1.0000
Schober's test	3.92 ± 1.75	4.39 ± 2.28	1.0000	4.88 ± 3.40	5.06 ± 1.96	1.0000

### Comparison of Effectiveness Indicators

**Figure 2:**
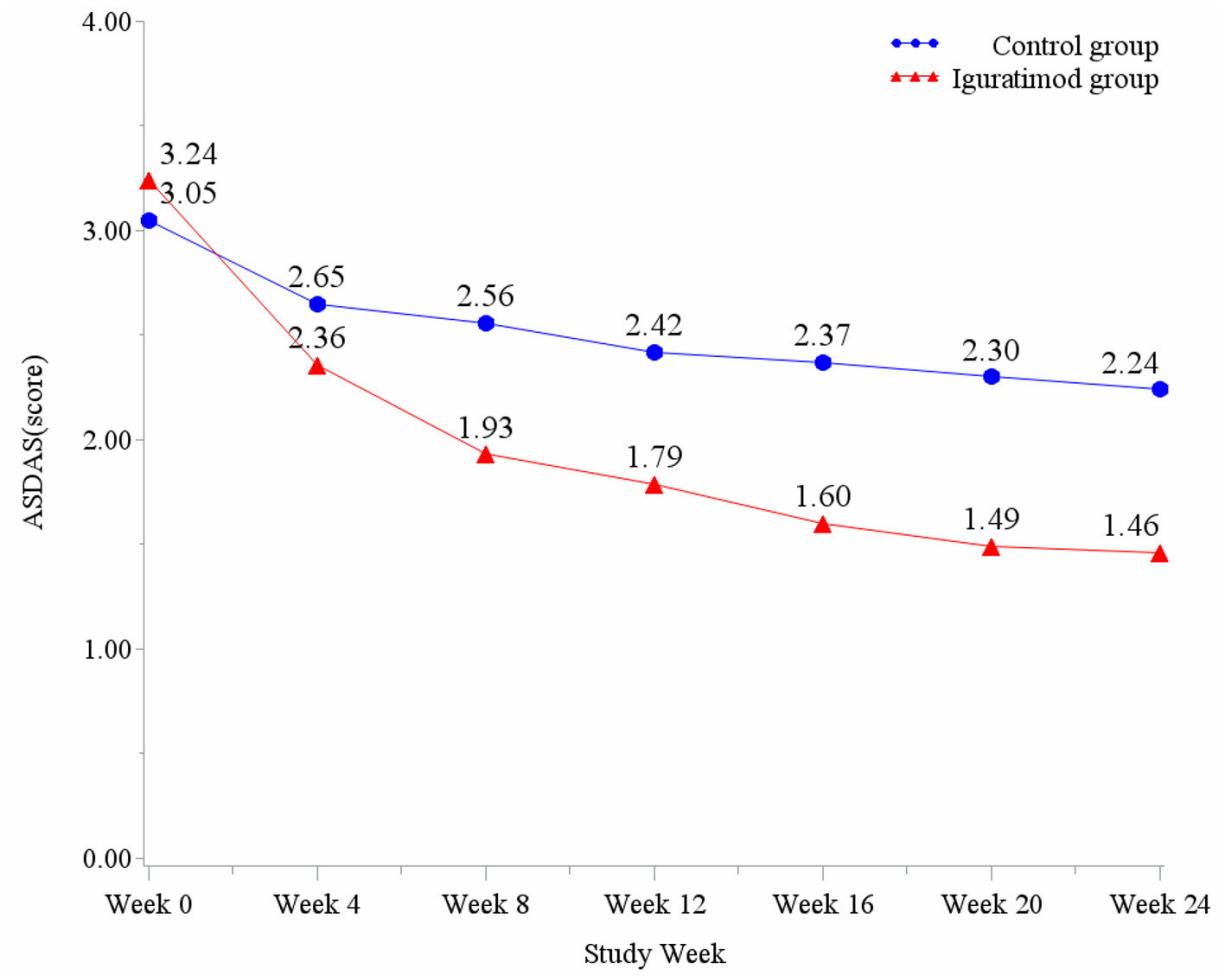
ASDAS change trend chart at 0–24 weeks.

**Figure 3:**
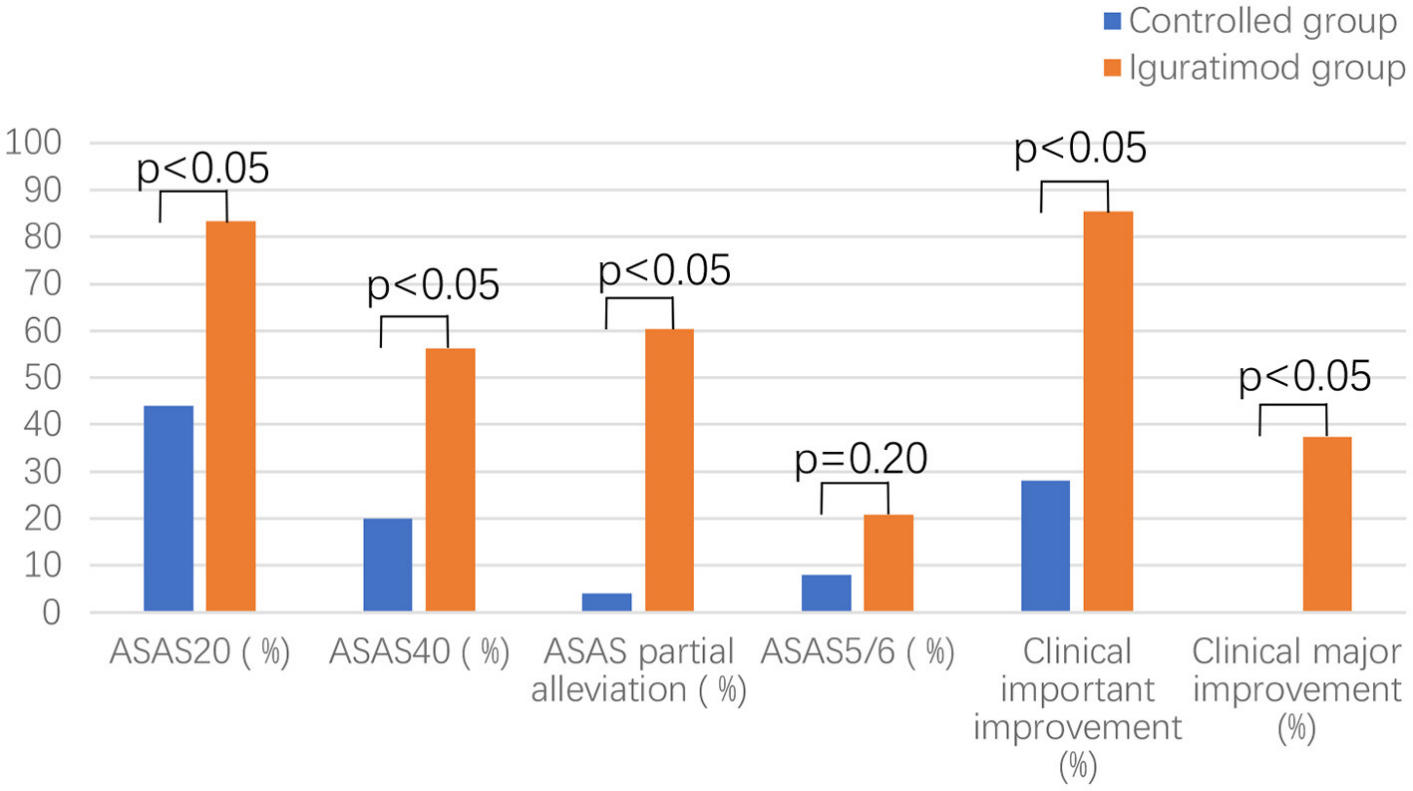
Comparison of effectiveness indicators between the two groups of patients after 24 weeks.

ASDAS change trend chart at 0–24 weeks is shown in Figure 2. The ratios of 12 and 24-week disease activity scores were compared to achieve the clinical important and major improvements between the two groups. The results are shown in Figure 3. The clinical important and major improvement rates in the Iguratimod group were higher than those in the control group after 12 and 24 weeks of treatment (*P* < 0.05). The two groups were compared to determine whether a difference in the proportion of ASAS20, ASAS40, ASAS5/6, and ASAS partial alleviation existed after 12 and 24 weeks. The results are shown in Figure 3. At the end of 24 weeks of treatment, the ASAS20, ASAS40, and ASAS partial alleviation rates in the Iguratimod group were higher than those in the control group (*P* < 0.05). The changes in BASDAI, BASFI, and BASMI from baseline between the two groups was compared after 12 and 24 weeks of treatment. The results are shown in Table 3. The difference in the improvement of BASDAI and BASFI between the two groups was statistically significant at 12 and 24 weeks (*P* < 0.05) but that of BASMI was not (*P* > 0.05). The following parameters were compared between the two groups: night back pain, total back pain after 12 and 24 weeks, the duration of morning stiffness, patient global assessment (PtGA), the physician global assessment (PGA), the number of peripheral swollen joints, the number of peripheral tender joints, tragus to wall distance, lateral flexion, modified Schober's, platelet, erythrocyte sedimentation rate, CRP, and other changes from baseline. The results are shown in Tables 2, 3. At the end of 12 weeks of treatment, the two groups had statistically significant differences from baseline in terms of night back pain, total back pain, the PtGA and the PGA from baseline (*P* < 0.05). The improvement range of the above indicators in the Iguratimod group was higher than that in the control group. At the end of 24 weeks of treatment, the two groups had statistically significant differences from baseline in terms of night back pain, total back pain, the PtGA, the PGA and CRP (*P* < 0.05). The improvement range of the above indicators in the Iguratimod group was higher than that in the control group.

**Table 3:** Comparison of effectiveness changes indicators between the two groups of patients after 12 and 24 weeks treatments (quantitative indicators).

**Parameters**	**Changes from baseline at 12 weeks**			**Changes from baseline at 24 weeks**		
	**CG (*n* = 25)**	**IG (*n* = 48)**	***P***	**CG (*n* = 25)**	**IG (*n* = 48)**	***P***
BASDAI	1.24 ± 1.20	2.59 ± 1.20	< 0.0001	1.52 ± 1.10	3.36 ± 1.17	< 0.0001
BASFI	0.83 ± 0.99	1.41 ± 0.77	0.0079	1.43 ± 1.26	2.26 ± 1.16	0.0066
BASMI	0.56 ± 1.04	0.44 ± 1.01	0.6543	0.52 ± 1.08	0.65 ± 1.23	0.9171
Night back pain	1.60 ± 1.41	3.88 ± 1.59	< 0.0001	2.48 ± 1.45	4.94 ± 1.60	< 0.0001
General back pain	0.92 ± 1.19	2.75 ± 1.28	< 0.0001	1.40 ± 1.53	3.85 ± 1.34	< 0.0001
PtGA	1.36 ± 1.55	3.23 ± 1.31	< 0.0001	2.12 ± 1.30	4.27 ± 1.35	< 0.0001
PGA	1.08 ± 1.00	2.79 ± 1.17	< 0.0001	1.60 ± 1.26	4.00 ± 1.11	< 0.0001
Platelet (× 10^9^/L)	11.16 ± 48.17	11.17 ± 43.47	0.9995	13.00 ± 58.27	22.40 ± 43.88	0.4415
ESR (mm/h)	7.68 ± 17.70	10.40 ± 15.60	0.5026	10.96 ± 19.85	11.85 ± 16.67	0.8393
CRP (ug/L)	2.48 ± 10.92	6.57 ± 7.80	0.0177	1.32 ± 11.42	7.78 ± 8.95	0.0022
Duration of morning stiffness (hours)	1.96 ± 2.76	3.13 ± 2.60	0.0797	2.48 ± 2.97	3.73 ± 2.61	0.0371
Lateral flexion	−0.44 ± 2.75	−1.41 ± 2.86	0.2114	−1.36 ± 3.68	−1.41 ± 2.94	0.5274
Tragus to wall distance	−0.12 ± 1.94	0.54 ± 1.93	0.3804	0.36 ± 1.98	0.81 ± 2.28	0.7593
Schober's test	−0.40 ± 1.04	−0.43 ± 1.36	0.6809	−0.96 ± 2.86	−0.68 ± 2.00	0.8407

### Comparison of the Quality of Work and Life Between the Two Groups Before Treatment and at the End of 24-Week Follow Up After Treatment

**Figure 4:**
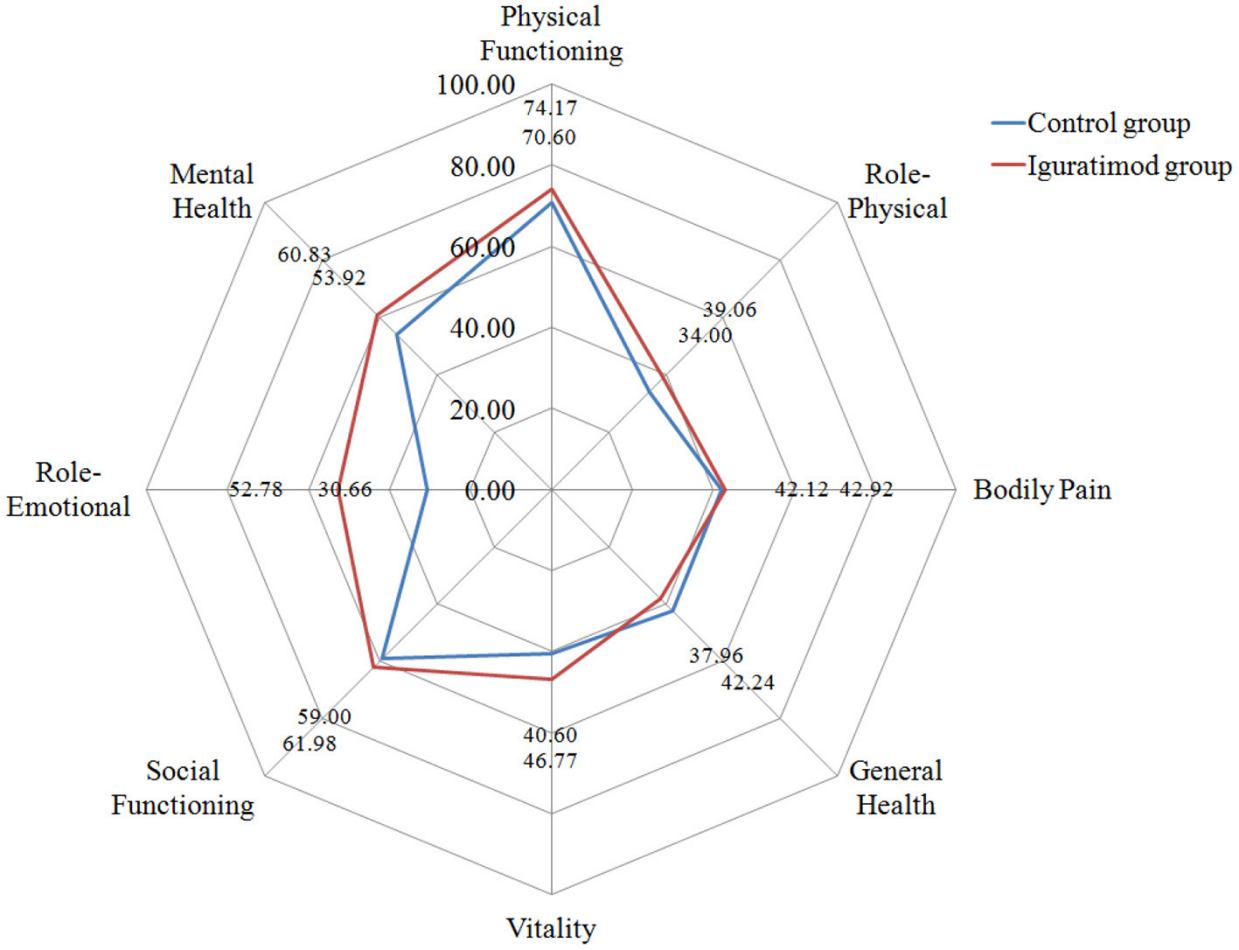
SF-36 radar chart at baseline (0 w).

**Figure 5:**
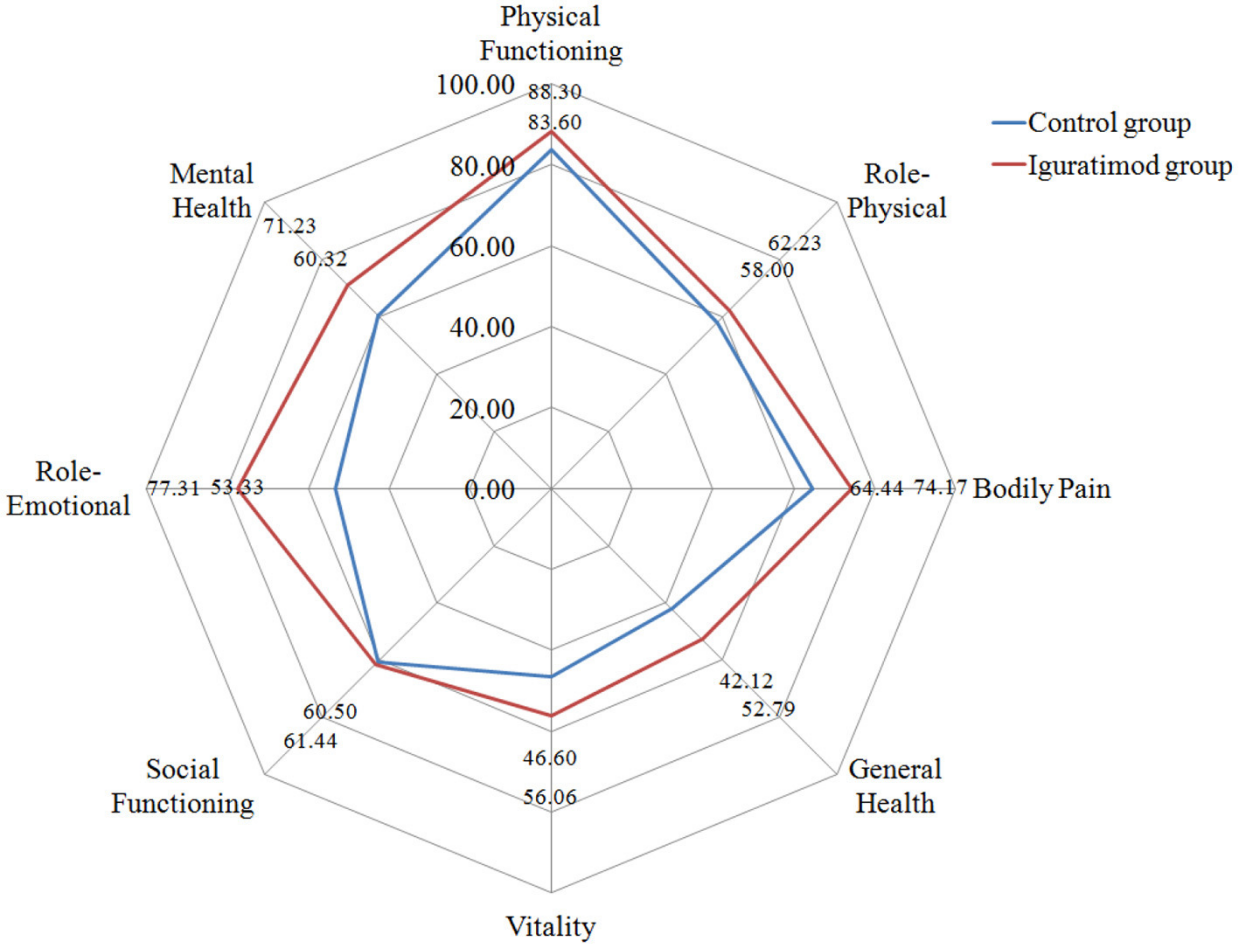
Radar chart of SF-36 after 24 weeks treatment.

Table 4 and Figures 4, 5 show the comparison results of the SF-36 scores between the two groups before treatment and at the end of 24 weeks of treatment. No statistically significant difference was observed in the eight dimensions of the SF-36 scale between the two groups before treatment (*P* > 0.05). After 24 weeks of treatment, the differences in scores on the four dimensions of physical pain, general health, energy, emotional function, and mental health were statistically significant (*P* < 0.05) and the score of the Iguratimod group was higher than that of the control group. Table 4 compares the effects of SPA on work and life between the two groups before treatment and at the end of 24 weeks of treatment. Before treatment, no significant difference was found in the eight items of SF-36 and the total score of ASASHI between the two groups. At the end of 24 weeks of treatment, statistically significant differences were observed in physical pain, mental health, health-related work productivity loss and the impact of health on daily life between the two groups.

**Table 4:** Comparison of quality-of-life indicators between the two groups before treatment and after 24 weeks of treatment.

**Parameters**	**Baseline**			**24 weeks follow-up**		
	**CG (*n* = 25)**	**IG (*n* = 48)**	***P***	**CG (*n* = 25)**	**IG (*n* = 48)**	***P***
Physiological function	70.60 ± 18.84	74.17 ± 15.38	0.3874	83.60 ± 15.24	88.30 ± 12.52	0.3979
Physiological job	34.00 ± 32.18	39.06 ± 40.58	0.7198	58.00 ± 38.68	62.23 ± 37.18	0.6602
Body pain	42.12 ± 16.98	42.92 ± 15.05	0.9158	64.44 ± 17.49	74.17 ± 16.19	0.0203
Health situation	42.24 ± 20.02	37.96 ± 14.73	0.6192	42.12 ± 26.68	52.79 ± 20.05	0.0653
Energy	40.60 ± 15.02	46.77 ± 19.83	0.1769	46.60 ± 20.40	56.06 ± 15.43	0.0228
Social function	59.00 ± 11.70	61.98 ± 14.80	0.2186	60.50 ± 4.68	61.44 ± 6.29	0.3913
Emotional function	50.66 ± 35.91	52.78 ± 41.74	0.0506	53.33 ± 45.14	77.31 ± 31.94	0.0317
Psychological status	53.92 ± 15.15	60.83 ± 17.72	0.0906	60.32 ± 18.44	71.23 ± 12.94	0.0139
ASAS HI total score	8.24 ± 4.25	6.88 ± 3.39	0.1069	4.68 ± 4.26	2.96 ± 2.97	0.1565
Health-related job productivity loss	0.38 ± 0.18	0.38 ± 0.20	0.9292	0.27 ± 0.14	0.15 ± 0.14	0.0005
Loss of productivity due to sickness	0.16 ± 0.32	0.15 ± 0.30	0.9024	0.01 ± 0.04	0.04 ± 0.17	0.4876
Total work productivity loss	0.48 ± 0.26	0.43 ± 0.23	0.5868	0.28 ± 0.15	0.16 ± 0.20	0.0007
The impact of illness on daily life	0.45 ± 0.21	0.43 ± 0.18	0.6270	0.28 ± 0.14	0.14 ± 0.12	0.0002

### Safety Evaluation

In this study, 12 cases of gastrointestinal discomfort occurred (all mild pain), of which eight (16.7%) were in the Iguratimod group and four (16.0%) belonged to the placebo group. No statistical difference was found between the two groups. For scientific significance, two cases underwent gastroscopy, one case showed superficial gastritis, no obvious ulcers, or bleeding was noted and one case showed gastric mucosa swelling. Three cases of elevated transaminase and one case of diarrhoea were found in the Iguratimod group but not in the placebo group. No significant difference was observed between the two groups. No clinical reports of gastrointestinal bleeding nor perforation were found, even cases of significantly lower or higher blood routine indices nor renal function damage.

## Discussion

Previous studies have found that TNF-α is closely related to AS and the IL-17/IL-23 axis may play a key role in the pathogenesis (8, 9) of this disease. TNF-α is an important pro-inflammatory cytokine, a key regulator of leukocyte adhesion molecules and a major stimulator of inflammatory cells. IL-17 is also a pro-inflammatory cytokine that could induce the activation of T cells and macrophages and the production of various pro-inflammatory mediators, such as IL-1, IL-6, and TNF. IL-17 and inflammatory factor TNF-α have a synergistic effect, whilst IL-17 could strengthen the inflammatory effect of TNF-α. Iguratimod is a new type of small molecule compound with anti-inflammatory and immunomodulatory effects. It could alleviate joint damage and immune abnormalities in chronic arthritis and autoimmune diseases; inhibit inflammatory cytokines (IL-1, IL-6, IL-8, and TNF) production; block the IL-17 signalling pathway and inhibit lymphocyte proliferation and immunoglobulin production (10). In terms of clinical manifestations, Iguratimod could significantly reduce the levels of ESR, C-reactive protein, immunoglobulin, IL-1, IL-6, TNF and other inflammation diagnostic parameters (7, 11). Moreover, in clinical studies, the safety and effectiveness of Iguratimod have been verified in patients with rheumatoid arthritis (12–14). However, strong evidence to support its use in patients with SpA is still lacking.

According to the knowledge of the authors, this trial is the first randomised, controlled trial to evaluate the efficacy and safety of Iguratimod on active SpA. The results showed that Iguratimod could improve the symptoms and signs of active spondylitis, thus providing evidence for the treatment of SpA by Iguratimod. Compared with the placebo, Iguratimod could effectively improve the patients' situation. In addition, Iguratimod was found to be safe and well-tolerated after 24 weeks of observation.

A prospective case study involving 17 cases of refractory axial SpA in China confirmed that T-614 has a good effect on disease activity and inflammatory markers in patients with active axial SpA who failed NSAIDs. Unlike those in the current study, these patients had poor tolerance to T-614. Eight (47%) patients discontinued the drug due to side effects, most of which were gastrointestinal diseases (15). Most patients considered in this study were refractory to SpA treatment. The medication was also more complicated; in addition to non-steroidal drugs, other DMARDs may be the main cause of poor tolerance. DMARDs have many adverse effects, amongst which gastrointestinal discomfort is the most common (16, 17). In the present study, eight cases (16.7%) in the Iguratimod group suffered from gastrointestinal discomfort, manifested as mild abdominal pain, and it disappeared after the proton pump inhibitor omeprazole was added. The elevated transaminases in three patients returned to normal after stopping the drug; this finding was consistent with that of other studies (18).

## Limitations

First, this study is a single-centre, small-sample, randomised controlled study. Although it meets the requirements of clinical research, the evidence is relatively weak. Multi-centre, large-sample, randomised controlled studies, and long-term follow up could be carried out in the future to further clarify the efficacy and safety of Iguratimod in patients with active SpA, especially its effects on other systems and organs. Second, due to the short period of the study, the patients were not provided with repeated imaging.

## Conclusion

Iguratimod has good efficacy and safety in patients with active SpA.

## Data Availability Statement

The raw data supporting the conclusions of this article will be made available by the authors, without undue reservation.

## Ethics Statement

The studies involving human participants were reviewed and approved by Ethics Committee of General Hospital of Chinese People's Liberation Army. The patients/participants provided their written informed consent to participate in this study.

## Author Contributions

FH, JZhu, and JiaZ designed the research. FH modified the paper. YL, KL, ZZ, YW, JG, JJ, and JieZ performed the research. KL and YL analysed data. YL wrote the paper. All authors contributed to the article and approved the submitted version.

## Conflict of Interest

The authors declare that the research was conducted in the absence of any commercial or financial relationships that could be construed as a potential conflict of interest.
