# Supplementary figures and images for: Randomised, Double-Blind, Placebo-Controlled Study of Iguratimod in the Treatment of Active Spondyloarthritis
Source: Front Med (Lausanne). 2021 Jun 2;8:678864. doi: 10.3389/fmed.2021.678864 (PMC8208078; doi:10.3389/fmed.2021.678864)

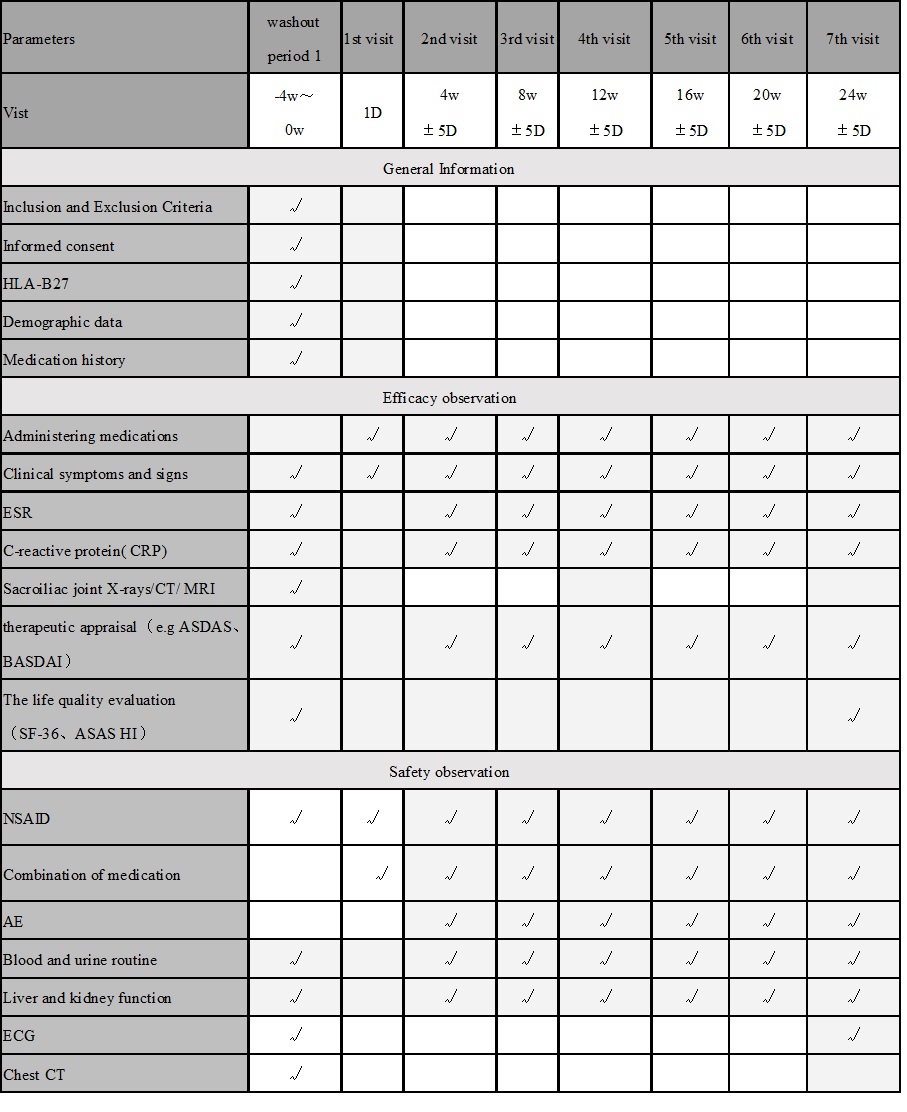

Supplement: Supplementary file 1 [file Image_1.jpg]
